# International Society of Sports Nutrition Position Stand: nutritional considerations for single-stage ultra-marathon training and racing

**DOI:** 10.1186/s12970-019-0312-9

**Published:** 2019-11-07

**Authors:** Nicholas B. Tiller, Justin D. Roberts, Liam Beasley, Shaun Chapman, Jorge M. Pinto, Lee Smith, Melanie Wiffin, Mark Russell, S. Andy Sparks, Lauren Duckworth, John O’Hara, Louise Sutton, Jose Antonio, Darryn S. Willoughby, Michael D. Tarpey, Abbie E. Smith-Ryan, Michael J. Ormsbee, Todd A. Astorino, Richard B. Kreider, Graham R. McGinnis, Jeffrey R. Stout, JohnEric W. Smith, Shawn M. Arent, Bill I. Campbell, Laurent Bannock

**Affiliations:** 1 Division of Pulmonary and Critical Care Physiology and Medicine, The Lundquist Institute for Biomedical Innovation at Harbor-UCLA Medical Center, Torrance, CA USA; 2 Academy of Sport and Physical Activity, Faculty of Health and Wellbeing, Sheffield Hallam University, Sheffield, UK; 3 Cambridge Centre for Sport and Exercise Sciences, School of Psychology and Sports Science, Anglia Ruskin University, Cambridge, UK; 4 School of Social and Health Sciences, Leeds Trinity University, Leeds, UK; 5 Sport Nutrition and Performance Research Group, Department of Sport and Physical Activity, Edge Hill University, Ormskirk, Lancashire UK; 6 Carnegie School of Sport, Leeds Beckett University, Leeds, UK; 7 College of Health Care Sciences, Nova Southeastern University, Fort Lauderdale, FL USA; 8 Department of Health, Human Performance, and Recreation, Baylor University, Waco, TX USA; 9 Department of Physiology, Brody School of Medicine, East Carolina University, Greenville, NC USA; 10 Department of Exercise and Sport Science, University of North Carolina, Chapel Hill, NC USA; 11 Institute of Sports Sciences & Medicine, Department of Nutrition, Food and Exercise Sciences, Florida State University, Tallahassee, FL USA; 12 Discipline of Biokinetics, Exercise and Leisure Sciences, School of Health Sciences, University of KwaZulu-Natal, Durban, South Africa; 13 Department of Kinesiology, California State University San Marcos, San Marcos, CA USA; 14 Department of Health & Kinesiology, Texas A&M University, College Station, TX USA; 15 Kinesiology and Nutrition Sciences, University of Nevada, Las Vegas, NV USA; 16 College of Health Professions and Sciences, University of Central Florida, Orlando, FL USA; 17 Department of Kinesiology, Mississippi State University, Mississippi, MS USA; 18 Department of Exercise Science, University of South Carolina, Columbia, SC USA; 19 Exercise Science Program, Performance & Physique Enhancement Laboratory, University of South Florida, Tampa, FL USA; 20 Institute of Performance Nutrition, London, UK

**Keywords:** Endurance, Nutrition, Performance, Racing, Supplementation, Training, Ultra-marathon

## Abstract

**Background**

In this Position Statement, the International Society of Sports Nutrition (ISSN) provides an objective and critical review of the literature pertinent to nutritional considerations for training and racing in single-stage ultra-marathon. ***Recommendations for Training****.* i) Ultra-marathon runners should aim to meet the caloric demands of training by following an individualized and periodized strategy, comprising a varied, food-first approach; ii) Athletes should plan and implement their nutrition strategy with sufficient time to permit adaptations that enhance fat oxidative capacity; iii) The evidence overwhelmingly supports the inclusion of a moderate-to-high carbohydrate diet (i.e., ~ 60% of energy intake, 5–8 g·kg^− 1^·d^− 1^) to mitigate the negative effects of chronic, training-induced glycogen depletion; iv) Limiting carbohydrate intake before selected low-intensity sessions, and/or moderating daily carbohydrate intake, may enhance mitochondrial function and fat oxidative capacity. Nevertheless, this approach may compromise performance during high-intensity efforts; v) Protein intakes of ~ 1.6 g·kg^− 1^·d^− 1^ are necessary to maintain lean mass and support recovery from training, but amounts up to 2.5 g.kg^− 1^·d^− 1^ may be warranted during demanding training when calorie requirements are greater; ***Recommendations for Racing***. vi) To attenuate caloric deficits, runners should aim to consume 150–400 Kcal·h^− 1^ (carbohydrate, 30–50 g·h^− 1^; protein, 5–10 g·h^− 1^) from a variety of calorie-dense foods. Consideration must be given to food palatability, individual tolerance, and the increased preference for savory foods in longer races; vii) Fluid volumes of 450–750 mL·h^− 1^ (~ 150–250 mL every 20 min) are recommended during racing. To minimize the likelihood of hyponatraemia, electrolytes (mainly sodium) may be needed in concentrations greater than that provided by most commercial products (i.e., > 575 mg·L^− 1^ sodium). Fluid and electrolyte requirements will be elevated when running in hot and/or humid conditions; viii) Evidence supports progressive gut-training and/or low-FODMAP diets (fermentable oligosaccharide, disaccharide, monosaccharide and polyol) to alleviate symptoms of gastrointestinal distress during racing; ix) The evidence in support of ketogenic diets and/or ketone esters to improve ultra-marathon performance is lacking, with further research warranted; x) Evidence supports the strategic use of caffeine to sustain performance in the latter stages of racing, particularly when sleep deprivation may compromise athlete safety.

## Background

Ultra-marathons are footraces that exceed the traditional marathon distance of 26.2 miles (42.2 km) [1, 2]. Participation has steadily increased in the last 30 years [3] and, despite its popularity as a competitive sport, most participants approach racing as a means of personal accomplishment [4]. Ultra-marathons are contested the world over, often in remote locations, on a variety of terrains, and in extremes of temperature and altitude. The nutritional demands of training and racing are congruent with the distances being contested, the latter of which is highly variable, for example: 31 miles/50 km (Blackwater Trail - Florida, USA); 56 miles/90 km (Comrades Marathon - Durban, South Africa); 100 miles/161 km (Western States Endurance Run - California, USA); and 152 miles/245 km (Spartathlon – Athens, Greece). Moreover, such races typically last between 6 and 48 h. The distances of multi-stage events can range from 150 miles/240 km (Marathon Des Sables - Sahara Desert, Africa) to 3100 miles/4989 km (Self-Transcendence 3100 - New York, USA); however, in order to permit more targeted recommendations, this Position Stand will focus on single-stage events up to and including 152 miles (245 km).

Nutrition is a critical component of the preparation phase and might influence the physiological adaptations to training via several means. Firstly, moderating carbohydrate (CHO) intake and aligning it with the flux in training volume and intensity may optimize endurance adaptations via the mediation of adenosine-5′-phosphate- (AMP-) activated protein kinase (AMPK) cell-signalling pathways [5]. Conversely, exercising while chronically glycogen-depleted increases circulating stress hormones (e.g., cortisol), and causes disturbances in several indices of immune function (e.g., circulating leukocytes) [6] thereby increasing susceptibility to overtraining. Secondly, in addition to meeting the requirements of glycogen resynthesis, optimal recovery is dependent on endurance athletes meeting their daily protein requirements [7]; this, in turn, will assist with muscle growth and/or maintenance. Thirdly, failing to adequately hydrate *during* training, and/or rehydrate *following* training, can result in carry-over effects that may reduce performance in subsequent sessions. Chronically, this can cause changes in vasopressin and markers of metabolic dysfunction or disease [8].

With respect to racing, runners must endure numerous physiological stresses (e.g., substrate depletion, dehydration, muscle damage, oxidative stress) which can have both acute and chronic health implications, and these can be partially addressed through nutritional interventions. For example, poorly-managed ultra-marathon hydration and electrolyte strategies can result in exercise-associated hyponatremia (serum sodium <135 mmol·L^− 1^), which is a potentially fatal complication of long-distance racing [9]. Moreover, offsetting dehydration can help slow the degradation of exercise [10] and cognitive performance [11] that is associated with a loss of body water. Long-duration exercise is also associated with a generalized inflammatory state, often characterized by immunosuppression, which can be partly assuaged by a well-balanced diet that provides the athlete with sufficient macro- and micronutrients [12].

A recent review [13] highlighted that although approximately 90% of amateur ultra-marathon runners consider nutrition to play a fundamental role in performance, many athletes still neglect basic empirical recommendations [14]. Indeed, while race completion has been positively correlated with energy and fluid intake [14, 15], the calories consumed by some ultra-endurance athletes is reported to be between 36 and 53% of their racing energy expenditure [13, 15–17]. Accordingly, by implementing nutritional strategies that are congruent with the physical stresses of training and racing, it may be possible to simultaneously optimize training adaptations, maximize race performance, and mitigate the negative consequences of race participation.

Despite the importance of sports nutrition for ultra-marathon training and racing, athletes and coaches face a number of obstacles in satisfying the nutritional demands, including: poor appreciation of the physiological demands of ultra-marathon; poor education (of coach/athlete/support staff) with respect to the nutritional demands of the sport; a high prevalence of athlete gastrointestinal (GI) distress; inconsistent food/fluid timing and rationing at checkpoints; the need to minimize pack-weight in self-sufficient races; placebo effects and confirmation bias from prior race experiences; the changes in food/fluid palatability associated with prolonged endurance exercise; sleep deprivation and extremes of temperature/altitude which are known to influence appetite [18–20]. Importantly, although ultra-endurance athletes have a reasonable knowledge of nutrition, they tend to favour the insights of other athletes over qualified nutrition experts [21]. Accordingly, the aim of this paper is to provide an accessible, evidence-based Position Stand on the nutritional considerations of ultra-marathon training and racing to inform best-practice of athletes, coaches, medics, support staff, and race organizers. This is particularly pertinent given the increased participation in ultra-marathon racing across the globe, and the ever-expanding extremes of race demands.

## Evidence statements

This Position Stand is concerned primarily with the nutritional considerations for single-stage ultra-marathon training and racing. Articles were searched via three online databases (Pubmed, MEDLINE, and Google Scholar), and the main search-terms comprised various combinations of the following: extreme-endurance, hydration, marathon, nutrition (various terms), pathophysiology, physiology, supplements (various terms), ultra-marathon, and ultra-endurance. The reference-lists of those articles selected for inclusion were manually searched for additional literature. The data informing our recommendations are incomplete, particularly relative to other sports, for several reasons. Firstly, despite the growing popularity of ultra-marathon, participant numbers are still relatively low. Moreover, runners are often reluctant to compromise their race preparation and/or recovery to volunteer for data-collection, particularly when invasive, time-consuming testing protocols are used. Secondly, ultra-marathons are often contested in remote locations and environmental extremes which do not lend themselves to complex or invasive data-collection protocols, especially when requiring equipment that is difficult to transport. For this review, therefore, the decision was made to include all published studies that were relevant to the topic, irrespective of any methodological concerns that may have arisen (e.g., low sample sizes, short study durations, lack of randomization, lack of control measures, and other biases). We have, nevertheless, been clear with respect to methodological limitations of the studies included. Furthermore, we have graded the strength of our evidence statements according to the system employed by the National Heart, Lung, and Blood Institute (NHLBI [22]), which we have adapted to incorporate a fourth level pertinent to case-reports. The system in question has also been used by other nutrition-related reviews [23]. Table 1 is a summary of the grading system and evidence categories.

**Table 1:** Grading system and evidence strategies

Evidence category	Sources of evidence	Definition
A	Meta-analyses, position-stands, and randomized-controlled trials (RCTs)	Evidence from meta-analyses, position stands, and well-designed RCTs (or trials that depart only minimally from randomization) that provide a consistent pattern of findings in the population for which the recommendation is made.
B	Systematic reviews including RCTs of limited number	Evidence from endpoints of intervention studies that include only a limited number of RCTs, post hoc or subgroup analysis of RCTs. In general, Category B is relevant when few randomized trials exist, they are small in size, and/or the trial results are somewhat inconsistent.
C	Nonrandomized trials/observational studies, other reviews (e.g., narrative)	Evidence from outcomes of uncontrolled/nonrandomized trials or from observational studies. Reviews that may harbour a specific narrative.
D	Case-reports	Evidence from low number or single-subject designs that report on unique observations or events, not necessarily applicable to broader populations.

## Considerations for training

### Energy and macronutrient demands

The foremost nutritional challenge facing the ultra-marathon runner is meeting the daily caloric demands necessary to optimize recovery and permit prolonged and repeated training sessions [24]. From a metabolic perspective, ultra-marathon racing places a heavy dependence on oxidative metabolism to utilize glycogen and fat stores efficiently; moreover, with increasing race distance, there is a substantial increase in the use of free fatty acids as fuel [25]. Therefore, a central aim of any periodized ultra-marathon training program should be to maximize capacity for fat metabolism, thereby sparing muscle glycogen for the latter stages of competition. Given that training volume and intensity will vary throughout the season, the energy and macronutrient intake must be periodized to accommodate variable training loads.

Daily caloric requirements are influenced by numerous factors, including: basal/resting metabolic rate [26], daily activity [27], specific training requirements, body composition, and thermogenesis that results from food digestion. The caloric demands of training will be further dependent on body mass (particularly lean mass), trained status, session distance/duration, and environmental terrain and conditions. Table 2 offers generalized estimates on the daily caloric requirements of ultra-marathon runners with respect to sex, session duration and pace, and the typical body mass/body fat extremes of ultra-marathon runners. The values presented are based on data from empirical studies [28, 29], and corroborated by independent reports suggesting that the energy cost of running ranges from 200 to 300 kJ·km^− 1^ (47–71 Kcal·km^− 1^) [30, 31]. As an example, a 50 kg female with 15% bodyfat, engaging in continuous running for 1 h·d^− 1^ (at a pace of 11.5 min·mile^− 1^; 8.4 km·h^− 1^) will require an estimated total of ~ 2004 Kcal·d^− 1^ in order to maintain caloric balance. The same athlete undertaking 3 h training sessions at the same pace would require ~ 2726 Kcal·d^− 1^, whereas a 3 h session performed at a pace of 7 min·mile^− 1^ (13.8 km·h^− 1^) would necessitate a considerably higher energy intake (i.e., ~ 3423 Kcal·d^− 1^) (Table 2). Training on challenging, variable, and uneven terrain, and in extremes of temperature and/or altitude, will notably increase the caloric and CHO requirements.

**Table 2:** Estimated daily caloric requirements for ultra-marathon runners, based on sex, typical extremes of body mass/fat, and session duration/pace

PACE	FEMALE				MALE			
	50 kg (15% BF)		70 kg (24% BF)		65 kg (10% BF)		85 kg (20% BF)	
	1 h	3 h	1 h	3 h	1 h	3 h	1 h	3 h
11.5 min·mile^− 1^ (8.4 km·h^− 1^)	2004	2726	2443	3455	2553	3492	2959	4187
9 min·mile^−1^ (10.7 km·h^− 1^)	2103	3023	2581	3870	2681	3878	3127	4692
7 min·mile^−1^ (13.8 km·h^− 1^)	2236	3423	2768	4430	2855	4398	3354	5372

e.g., a female runner of body mass 50 kg (~ 15% body fat), training for 1 h per day at a pace of 9 min·mile^− 1^, would need an estimated 2103 Kcal·d^− 1^. *BF* body fat. *h* hour

Careful consideration of the weekly requirements of both training and recovery is recommended to achieve energy balance, unless there is an individual goal of weight loss or gain. In addition, when nutritional intake cannot be matched (e.g., on heavy training days or following several bouts of exercise in short succession), energy intake above maintenance calories may be warranted on recovery days.

With respect to total energy intake, a macronutrient distribution of 60% CHO, 15% protein, and 25% fat is typically recommended to support repeated bouts of endurance training [32]. When expressed relative to body mass, ultra-marathon runners undertaking frequent bouts of intense training (e.g., 2–3 h·d^− 1^, 5–6 times per week) typically need ~ 5–8 g·kg^− 1^·d^− 1^ of CHO (for review, see [33]). For runners with greater training mileage and/or pace, carbohydrate intakes ranging from 7 to 10 g kg^− 1^·d^− 1^ may be warranted, pending the athlete’s metabolic flexibility (i.e., their individual capacity to readily switch between fat or CHO oxidation at high absolute work-loads [34]) and, specifically, their capacity to metabolize fat. With respect to macronutrient breakdown, Table 3 provides estimated daily requirements for individuals completing training runs at 11.5 min·mile^− 1^ (8.4 km·h^− 1^). Based on nitrogen-balance methodology, protein intakes of > 1.6 g·kg^− 1^·d^− 1^ have been recommended for endurance athletes who have high training demands [35]. However, for athletes with greater caloric requirements, relative protein intakes up to 2.5 g·kg^− 1^·d^− 1^ may be warranted. Unless strategically targeting a ketogenic approach, fat intakes ranging from 1.0–1.5 g·kg^− 1^·d^− 1^ are likely sufficient, although heavier/faster individuals may need fat intakes close to 2.0 g·kg^− 1^·d^− 1^ to support caloric needs.

**Table 3:** Estimated daily macronutrient requirements for ultra-marathon runners, based on sex, typical extremes of body mass/fat, and session duration/pace

	FEMALE				MALE			
	50 kg		70 kg		65 kg		85 kg	
	(15% BF)		(24% BF)		(10% BF)		(20% BF)	
	1 h	3 h	1 h	3 h	1 h	3 h	1 h	3 h
Carbohydrate (g·d^− 1^)	301	409	366	518	383	524	444	628
Carbohydrate (g·kg^− 1^·d^− 1^)	6.0	8.2	5.2	7.4	5.9	8.1	5.2	7.4
Protein (g·d^− 1^)	75	102	92	130	96	131	111	157
Protein (g·kg^− 1^·d^− 1^)	1.5	2.0	1.3	1.9	1.5	2.0	1.3	1.8
Fat (g·d^− 1^)	56	76	68	96	71	97	82	116
Fat (g·kg^− 1^·d^− 1^)	1.1	1.5	1.1	1.4	1.1	1.5	1.0	1.4
Energy Intake (Kcal·d^−1^)	2004	2726	2443	3455	2553	3492	2959	4187
Energy Intake (Kcal·kg^−1^·d^−1^)	40.1	54.5	34.9	49.4	39.3	53.7	34.8	49.3

e.g., a female runner with body mass 50 kg and 15% body fat, training for 1 h per day will need an estimated 301 g carbohydrate, 75 g protein, and 56 g fat. Overall values are based on 11.5 min·mile^− 1^ (8.4 km·h^− 1^) pace. *BF* body fat

#### Evidence statement (category A/B)

Nutritional strategies should be individualized and will be dependent on trained status, basal/resting metabolic rate, daily activity, specific training requirements, body composition, thermogenesis that results from food digestion, session distance/duration, and environmental terrain/conditions.

#### Evidence statement (category B/C)

The current evidence supports the contention that a macronutrient distribution of 60% CHO (7–10 g·kg^− 1^·d^− 1^), 15% protein (1.3–2.1 g·kg^− 1^·d^− 1^), and 25% fat (1.0–1.5 g·kg^− 1^·d^− 1^) is necessary to support repeated bouts of endurance training. However, differences among athletes with respect to training duration, pace, and body mass, will lead to a range of caloric requirements (for both males and females) from ~ 38–63 Kcal·kg^− 1^ d^− 1^.

### Nutrition to maximize fuel efficiency

#### Carbohydrate ingestion before training

The aim of ultra-marathon training should be to maximize fat metabolism in order to preserve muscle glycogen; therefore, nutrition strategies that promote or optimize fat oxidation should be prioritized. Carbohydrate pre-fuelling (within 90 min of session commencing), particularly with high-glycaemic foods, should be avoided due to a CHO-mediated insulin secretion from pancreatic ß-cells which supresses adipose tissue lipolysis [36]; this, in turn, may be counterproductive to the goals of ultra-marathon training. Pre-exercise CHO intake also facilitates the uptake of blood glucose into muscle, and suppresses hepatic (liver) glycogenolysis [37], which may increase the potential risk of hypoglycaemia during the early period of a training session in susceptible individuals [38], although any negative impact of this on short-duration exercise performance has been refuted [39]. Others have reported hypoglycaemia-like symptoms during exercise that follows CHO intake [40] which may negatively impact athlete effort perceptions. Collectively, these data support the notion that athletes should aim to commence training in a euglycemic state [41].

#### Train-low, compete-high

The contemporary guidelines suggest that endurance athletes should consume approximately 60% of their daily calories from CHO, aiming for 5–12 g·kg^− 1^·d^− 1^, depending on whether the daily exercise duration is *moderate* (~1 h per day) or *very high* (> 4 h per day) [42]. These daily intakes are deemed necessary to restore muscle and liver glycogen, satisfy the metabolic needs of the muscles and central nervous system, and ensure CHO availability for days of successive training. Nevertheless, a joint proposition from the Academy of Nutrition and Dietetics, Dietitians of Canada, and the American College of Sports Medicine [42] suggested that:

*“In some scenarios, when the focus is on enhancing the training stimulus or adaptive response, low carbohydrate availability may be deliberately achieved by reducing total carbohydrate intake, or by manipulating carbohydrate intake related to training sessions (e.g., training in a fasted state, undertaking a second session of exercise without adequate opportunity for refuelling after the first session)*.”

The notion of *train-low, compete-high* is based on insights from cellular biology suggesting that careful manipulation of glycogen via dietary CHO restriction can serve as a regulator of metabolic cell-signalling, which can optimize substrate efficiency and endurance adaptations [5]. This may be particularly beneficial in the early stages of a training regimen, thereby allowing sufficient time for adaptations to occur. Periodically training with low muscle glycogen is associated with the activation of signalling pathways, including AMPK, which play a crucial role in mitochondrial biogenesis. Importantly, this regulates key transporter proteins including glucose transporter-4 (GLUT-4) and the monocarboxylate transporters, both of which mediate endurance performance (for review, see [5]). Chronic training with lowered (but not depleted) glycogen stores can result in adaptations that, following glycogen resynthesis, increase total work and time to exhaustion during exercise [43]. In practice, training with lowered glycogen stores can be achieved by: i) *fasted sessions* [44] whereby low-to-moderate intensity training runs are completed in the morning before breakfast, given that liver glycogen stores are reduced by as much as 80% following an overnight fast [42]; ii) *low glycogen sessions* [44] whereby athletes intermittently exercise twice daily every second day, instead of training once daily, which may enhance gene transcription associated with fat oxidation [43, 45].

#### Consequences of carbohydrate restriction

The above-mentioned strategy has been scarcely studied in relation to ultra-marathon training and should, therefore, be practiced tentatively. Indeed, safe implementation requires nutrition-specific knowledge, an understanding of training periodization, and a degree of experience and self-awareness on behalf of the athlete with respect to their requirements. As such, athletes are cautioned against training in a *chronically* depleted state (especially during intensive training periods, or when repeated days of prolonged training are scheduled) as this may lead to low energy availability and, ultimately, relative energy deficiency (RED-S [46];). A further consideration is that high-intensity performance will likely be compromised by low glycogen availability, due to a relative inability to sustain a high work rate [45]. Exercising while glycogen-depleted increases circulating cortisol and causes disturbances in several indices of immune function (including plasma glutamine and circulating leukocytes) [6], and post-exercise immune dysfunction is most pronounced following prolonged, continuous exercise (> 1.5 h) performed without food [47]. As training volume and/or intensity increase (e.g., an increase in running mileage or a transition to interval training), relatively greater amounts of dietary CHO will be required to fuel performance and minimize the risk of injury. Consequently, before implementing a new dietary regimen, athletes and coaches must consider each individual’s metabolic needs, ideally having sought advice from a qualified nutrition professional, with the program monitored and adjusted based on the individual response. The practice of periodic CHO *moderation* should, therefore, be preferred to *restriction*.

#### High-fat, ketogenic diets

Another approach in modifying macronutrient intake to shift metabolic flexibility in favor of fat oxidation is the use of ketogenic diets. These have traditionally involved dramatic alterations in dietary fat utilizing a 4:1 fat:protein or fat:carbohydrate ratio. Modified ketogenic diets (70% of energy intake from fat) are also reported to increase fat metabolism [48], but may be more sustainable relative to traditional ketogenic approaches. The term *keto-adapted* has been used to denote a metabolic shift towards efficient use of ketone bodies. While debate exists, keto-adaptation may take several weeks or months, indicating that sustained tolerance to high-fat intake may be necessary in order that the individual acquire the full benefits.

Various ketogenic strategies have been studied (e.g., cyclical, intermittent fasting) with the premise of increasing ketone production and subsequent oxidation (i.e., nutritional ketosis ~ 0.5–3.0 mmol·L^− 1^). Early studies in endurance-trained athletes demonstrated potential ergogenic effects of a short-term ketogenic diet [49], but have been criticized due to low participant numbers (*n* = 5), with poor consideration of individual responses and negligible performance gains. More importantly, such studies may not be applicable to training durations typical of ultra-marathon (> 2.5 h). Nevertheless, ketogenic diets have been shown to reduce muscle glycolysis [50] and may, therefore, be useful during ‘adaptive’ periods of training to facilitate a rapid metabolic shift towards fat oxidation, resulting in decreases in body mass. In a group of ultra-marathon runners performing 3 h of submaximal treadmill running, a prior ketogenic diet resulted in fat oxidation rates of ~ 1.2 g·min^− 1^ which were significantly higher than that observed in subjects who had followed a high CHO diet (~ 0.75 g·min^− 1^) [48]. However, the subsequent impact of this change in substrate efficiency on exercise performance is unclear. Although early research into ketogenic diets proposed a CHO upper-limit of 50 g·d^− 1^, Volek et al. [48] reported improved substrate efficiency during exercise when athletes followed a less conservative CHO intake (80 g·d^− 1^). Accordingly, a *strict* ketogenic diet may not be necessary to promote fat oxidation in ultra-marathon runners.

Notwithstanding the available research which indicates a degree of benefit, ketogenic diets have been associated with acute negative symptoms, including: fatigue, headaches, poor concentration, lethargy, GI discomfort, nausea, and weight loss. All such symptoms may have consequences for training, particularly when resulting in immunosuppression and decreases in lean mass. Furthermore, it is plausible that runners training in a glycogen-depleted state, and who are insufficiently *keto-adapted*, may become acutely catabolic. It should also be noted that significant increases in fat intake are often congruent with decreased intake of fiber and micronutrients (specifically, iron, magnesium, potassium, folate, and antioxidants) [51]. Previous studies into sustained ultra-endurance exercise have highlighted concerns with decreased intakes of some micronutrients (magnesium and B-vitamins [52, 53]) and, as such, a mineral-rich approach involving plant-based foods and wholegrains should be incorporated into the overall nutrition strategy to support broader training requirements.

Finally, available data support the contention that while ketogenic approaches may enhance fuel utilization to favor fat oxidation, the ability to perform at higher intensities may be compromised, or even reduced, due to downregulation of pyruvate dehydrogenase [54], leading to reduced oxygen economy [55]. Despite positive anecdotal reports from ultra-marathon runners, there is insufficient literature to support the notion that sustained ketogenic diets are beneficial for performance, and caution is urged if following such a practice, especially when considering the influence of in-task CHO intake on substrate use during exercise.

#### Evidence statement (category B)

Strategically moderating CHO intake can facilitate metabolic adaptations associated with enhanced endurance performance. However, caution is advised against training chronically glycogen depleted, particularly during periods of repeated high-intensity exercise or prior to racing.

#### Evidence statement (category B/C)

Despite the use of ketogenic diets to facilitate a rapid metabolic shift towards greater fat oxidation, there is insufficient evidence to support the use of such diets in ultra-marathon training, and further research is warranted.

### Protein and muscle damage

Prolonged or strenuous exercise, particularly that to which the individual is unaccustomed, can result in muscle damage attributed to metabolic overload and/or mechanical strain [56]. Moreover, nitrogen balance can remain below baseline for several days following unaccustomed exercise [57]. The substantial training distances of ultra-marathon are associated with high levels of mechanical stress. This is reinforced by empirical data showing that whole-blood markers of muscle breakdown (e.g., creatine kinase, lactate dehydrogenase, and serum creatine phosphokinase) were higher following ultra-marathons when compared to marathons run at a relatively faster pace [58, 59]. Specifically, creatine kinase concentrations of 274 ± 71 U·L^− 1^ were observed post-marathon, relative to 2983 ± 1716 U·L^− 1^ following a 100 km race, and 4970 ± 2222 U·L^− 1^ after a 308 km race [58]. These data suggest that race distance and/or duration mediate muscle damage more than race intensity, although duration is not the sole determinant of muscle damage during ultra-marathon [60]. The environmental terrain typical of ultra-marathon also deserves consideration in the training program. Downhill running (on mountainous or undulating paths) is associated with greater peak flexion angles relative to level or uphill running; this exaggerates the eccentric component of impact-loading, thereby increasing muscle damage [56]. Indeed, muscle damage resulting from a single bout of downhill running can result in a shortened stride-length in subsequent efforts [61], and this may be pertinent for runners training on consecutive days.

Some authors suggest that the muscle damage and metabolic stress associated with 100 km ultra-marathons, and equivalent exhaustive efforts, represent a danger to human health [62], causing possible hepatic damage which warrants further study [60]. As such, although prior conditioning of the musculoskeletal system is critical for successful participation in ultra-marathon, participants should be mindful of nutritional strategies which may mitigate muscle damage and the associated inflammation during the training period. Satisfying metabolic demand for protein is, therefore, a prerequisite for both recovery and general health.

#### Protein dose and timing

Contemporary guidelines for athletes engaged in chronic endurance training suggest dietary protein in the amount of 1.2–2.1 g·kg^− 1^·d^− 1^ in order to support positive nitrogen balance and metabolic requirements [42, 63]. Current evidence indicates that protein intakes of less than 1.6 g·kg^− 1^·d^− 1^ may result in a negative nitrogen balance in endurance athletes who have high training demands [35]. Furthermore, amounts exceeding 2.1 g·kg^− 1^·d^− 1^ are unlikely to have additive effects on muscle protein synthesis, although the protein contribution to energy metabolism (and other structural/functional processes) may be greater in ultra-marathon runners engaged in very high-mileage training. This may, in turn, necessitate slightly higher intakes [64]. Higher protein amounts are also required when CHO and/or caloric intakes are low or insufficient [65]. A 20 g bolus of whey protein appears sufficient to maximize fractional synthetic rate after resistance exercise [66], with up to 30 g appropriate for larger athletes (>85 kg). Runners should also be mindful that protein needs may be higher in older adults [67, 68]. With respect to timing, an intermediate protein feeding strategy (~20 g every 3 waking hours) is more effective at stimulating muscle protein synthesis than pulse-feeding (~10 g every 1.5 h), or bolus-feeding (~40 g every 6 h) [69]. During chronic training, protein ingested before sleep appears to be an effective strategy to increase muscle protein synthesis overnight (for review, see [70]). Ultra-marathon runners who struggle to meet their protein needs through dietary means might choose to supplement, perhaps using whey protein due to its high bioavailability and complete amino acid profile [63].

#### Selected amino acids

The branched-chain amino acids (BCAAs) have been the focus of study for many years. An acute bout of prolonged exercise increases the rate of BCAA oxidation in skeletal muscle [71], suggesting that demands in ultra-marathon runners may be greater, but chronic training significantly attenuates the absolute rate of BCAA oxidation during exercise [71]. Therefore, the primary utility of BCAAs may be in muscle recovery and immune regulation during periods of hard training and racing [72, 73], particularly when consumed in the post-absorptive state [74]. Although meeting absolute protein demand is critical for the ultra-marathon runner, the literature suggests that L-leucine may support the upregulation of muscle protein synthesis, influencing mRNA translation and the mTOR cell-signalling pathway [75]. Although there are no existing studies on the efficacy of L-leucine specifically for ultra-marathon runners, there are reports that a 3–6 g daily dose of L-leucine might be beneficial for those engaged in strenuous endurance and/or resistance training [75]. Furthermore, L-leucine (5g) consumed with a small amount of whey protein (6g) may be as effective at stimulating muscle protein synthesis as a 25 g bolus of whey protein, although the latter may be more practical [76].

#### Evidence statement (category B/C)

Protein intakes of ~ 1.6–2.1 g·kg^− 1^·d^− 1^ are sufficient to optimally simulate muscle protein synthesis, which will likely support recovery from training. Intakes of up to 2.5 g·kg^− 1^·d^− 1^ may be warranted during demanding training periods (when caloric requirements may be substantially greater), or when CHO/caloric intake is insufficient.

#### Evidence statement (category B)

An intermediate protein feeding strategy of ~20 g every 3 waking hours may provide an optimal strategy to stimulate muscle protein synthesis for ultra-marathon runners.

### Daily hydration guidelines

A typical training session for the ultra-marathon runner appears sufficient to cause substantial dehydration. Over the half-marathon distance (13.1 miles), mean sweat losses of ~1.4 L were observed in male runners and, when offset against fluid ingestion during exercise, resulted in net fluid losses of ~ 0.3 L [77]. Over longer training distances (marathon), high-level runners exhibited a body weight loss of 0.3 and 1.7%, in cool and warm conditions, respectively, even when consuming fluid at a rate of 1 L·h^− 1^ [78]. Furthermore, abstaining from fluid resulted in an average dehydration of 3.3 and 5.3%, respectively [78]. Notwithstanding the commonly-reported effects of mild dehydration on subsequent exercise performance, chronic dehydration can influence health outcomes, with several authors noting dehydration-mediated changes in vasopressin, and markers of metabolic dysfunction or disease [8]. To mitigate carry-over effects from one session to the next, and to maintain general health, there are two components of hydration that warrant consideration in the periodized nutrition program: 1) hydration strategies to facilitate post-exercise recovery; and 2) day-to-day hydration requirements that are independent of training.

#### Post-exercise fluid intake

When recovery time is short, or the extent of fluid loss is great, thirst-driven fluid intake is not adequate to restore water balance [79]. Targeted fluid replacement strategies are, therefore, critical to maximize recovery before a subsequent session. It stands to reason that runners should replenish the fluid volume lost in training; this can be estimated via pre- to post-exercise body mass weighing. However, even in a hypohydrated state, the obligatory excretion of metabolic waste products allows for continued fluid losses [80]. Consequently, a fluid volume *greater* than that lost in training is necessary to fully restore water balance. This notion has been demonstrated empirically by both Shirreffs et al. [80] and Mitchell et al. [81], who reported that a low-sodium drink consumed at a volume of 150% of exercise-induced body mass loss resulted in enhanced hydration relative to an identical concentration consumed at 100% body mass loss. Greater fluid volumes up to 200% body mass loss may only lead to greater post-exercise hydration when consumed with higher concentrations of sodium (61 mmol·L^− 1^; 1403 mg·L^− 1^) [80], but fluid volumes above this are not recommended. As these data indicate, plain water is not likely sufficient to restore fluid balance following training due to the consequent decrease in plasma sodium concentration and osmolality [82] causing diuresis. Unequivocally, post-exercise urine output decreases as the drink sodium concentration increases; sodium intake should, therefore, ideally equal the concentration of sodium lost in sweat. The sodium content of commercial sports drinks (~20–25 mmol·L^− 1^; 460–575 mg·L^− 1^) is lower than that typically lost in sweat [83, 84] and should, therefore, be considered a conservative target. There is little research on the suggested *rate* of fluid intake, but the available data indicate that slow consumption (i.e., over several hours) will maximize the effectiveness of a rehydration strategy.

#### Day-to-day fluid intake

The actual fluid intake necessary to attain euhydration on a day-to-day basis will vary with renal and extrarenal water losses [85]; moreover, the absolute daily fluid intake (from food and drink) will vary widely among individuals. There are also daily fluctuations in total body water, estimated by Cheuvront et al. to have an upper-limit of ±1% of body weight (i.e., 0.6–0.9 kg in an adult of 60–90 kg) [86]. Interestingly, using biochemical measures of blood and urine, average plasma osmolality was found to be similar between groups of low-volume (1.2 L·d^− 1^) and high-volume (2–4 L·d^− 1^) drinkers [8]; it is possible, therefore, to attain euhydration with a range of fluid intakes. Indeed, elite Kenyan endurance runners have been shown to exhibit a euhydrated state when consuming fluid ad-libitum [87]. Moreover, given the sensitivity and reliability of the human thirst sensation to denote dehydration [79], it is reasonable to suggest that drinking-to-thirst is appropriate for responding to daily hydration needs. There are individuals with relatively high plasma osmolality thresholds for thirst [88], which can lead to chronic deviations from a euhydrated state. Accordingly, the thirst sensation may only be appropriate in instances of acute dehydration. For the ultra-marathon runner, hydration monitoring strategies are recommended (see *Hydration monitoring strategies*). In addition, overuse of fluids that contain insufficient concentrations of electrolytes (e.g., water or hypotonic sports drinks) may cause overhydration, decreased electrolyte concentrations, an increased risk of dilutional hyponatremia, and/or failure of the renal system [89] in extreme cases. Ultra-marathon runners are, therefore, cautioned against excessive fluid intakes to placate pseudoscientific claims that high fluid volumes are needed to ‘flush the kidneys’ or ‘remove toxins from the blood’.

#### Hydration monitoring strategies

Only an estimated 20% of endurance runners monitor their hydration status [90]. Although direct measures such as urine osmolality are rarely practical for most individuals, there are several simple and accessible tools that can be used to estimate hydration status. The urine color chart is the most common means of estimating hydration status in runners [90]. This simple technique involves the periodic assessment of urine color, whereby ‘pale-straw’ would indicate that the individual is well-hydrated (assuming this is not measured post-ingestion of a large bolus of fluid). The Venn diagram proposed by Cheuvront and Sawka [91] is a more sophisticated tool (appropriate for healthy, active, low-risk populations) which estimates hydration status by combining measures of nude body mass, thirst perception, and urine color.

#### Evidence statement (category B/C)

General day-to-day hydration can, in most instances, be achieved by following a drink-to-thirst (ad libitum) strategy.

#### Evidence statement (category A/B)

To inform post-training rehydration strategies, athletes should measure pre- to post-exercise body mass losses, and monitor their hydration status.

#### Evidence statement (category A/B)

After key training sessions, ingesting a fluid volume greater than that lost (150%) is necessary to restore water balance. Simultaneously, at least 460 mg·L^− 1^ of sodium should be ingested, either in food or as a supplement.

## Considerations for racing

### Energy and macronutrient demands

#### Energy expenditure

Given the durations typical of ultra-marathon, it is not feasible to meet caloric demands in their entirety. Several scenarios can be examined to reinforce this hypothesis. First, consider that a 50 kg athlete undertaking a 50 mile (80 km) race at 8.0 km·h^− 1^ (~ 10 h) will expend ~ 3460 Kcal. For the same event contested at the same pace, a 70 kg athlete would expend ~ 4845 Kcal (an approximate Kcal range of 346–484 Kcal·h^− 1^). Second, a 50 kg athlete undertaking a 100 mile (161 km) ultra-marathon at an average pace of 6.5 km·h^− 1^ may expend ~ 6922 Kcal in ~ 25 h, whereas at the same pace, a 70 kg athlete would likely expend ~ 9891 Kcal (range of 277–395 Kcal·h^− 1^). These values are similar to the estimated energy expenditures of 200–300 kJ·km^− 1^ (47.8–71.7 Kcal·km^− 1^) reported elsewhere [31]. When offset against the energy intakes observed in a typical ultra-marathon, runners are likely to exhibit a net calorie loss [92]. Accordingly, in addition to implementing an in-race nutrition strategy, an effort should be made to minimize caloric deficits before and after the race, and should be considered part of the overall holistic approach. Indeed, CHO availability for racing can be maximized by adhering to a contemporary loading strategy (i.e., ~10 g·kg^− 1^·d^− 1^) in the 48 h leading into the event [42, 44], with care taken to avoid GI distress. On race-day, runners are advised to consume a familiar, easily-digestible pre-race meal, rich in low-glycemic index CHO, while avoiding food with high fat and/or fiber content to minimize gut discomfort during the race.

#### Energy intake

Field studies indicate that successful completion of ultra-marathon is generally associated with greater energy and fluid intake [14, 15], even when accounting for variations in performance time [15]. A nuance of the longer distance event is that the lower average work rate permits a faster rate of gastric emptying, which tends to be compromised only at exercise intensities > 70% maximal oxygen uptake (V̇O_2_max) [93]. Consequently, relative to shorter races contested at a higher intensity, ultra-marathon runners can usually accommodate greater energy intake and more calorie-dense foods to the level of individual tolerance [94].

There is variability with respect to the absolute rate of energy intake reported during racing, but a sensible range can be determined. In 213 runners contesting one-of-three race distances (44, 67, or 112 km; Ultra Mallorca Serra de Tramuntana; Spain), mean energy intake was 183 Kcal·h^− 1^, with no discernible difference among race distances [95]. By contrast, in longer races (100 mile, 161 km), caloric intakes of < 200 Kcal·h^− 1^ tended to result in race non-completion [15], with race finishers consuming a significantly greater number of hourly calories when compared to non-finishers (4.6 ± 1.7 versus 2.5 ± 1.3 Kcal·kg^− 1^·h^− 1^). These findings have been reported elsewhere under similar race conditions [92]. Moreover, elite runners contesting a series of sixteen 100 mile (161 km) ultra-marathons, reported average energy intakes of 333 ± 105 Kcal·h^− 1^ [96]. Greater caloric intakes may, therefore, be necessary for longer races to enable performance.

Based on previous estimates of energy expenditure during running, and the above-mentioned research, the ISSN recommends a caloric intake of ~ 150–300 Kcal·h^− 1^ for race distances up to and including 50 miles (~ 81 km) during which any caloric deficits may be better tolerated. By contrast, in longer races when the magnitude of caloric deficits is greater and less likely to be well-tolerated, higher intakes of ~ 200–400 Kcal·h^− 1^ are suggested. Where GI distress is an issue, transient reductions in energy intake to the lower-end of this range are reasonable, congruent with a reduction in race pace. However, persistent calorie intakes of < 200 Kcal·h^− 1^ are not recommended, and when nausea precludes this rate of intake, a degree of perseverance/stubbornness with respect to feeding (within tolerance levels) may be required. This may be particularly pertinent in the latter stages of a race in order to minimize the risk of hypoglycaemia which can result in race non-completion, and reinforces the importance of progressive gut training during the preparation phase [97].

#### Carbohydrate versus fat intake

The mechanistic link between glycogen depletion in skeletal muscle and liver, and a subsequent early-onset fatigue during prolonged exercise was made in the 1960s [98]. In addition to negatively impacting endurance performance, the reduction in plasma glucose concentration that follows glycogen depletion is associated with acute cognitive decline; this, in turn, can compromise athlete safety on ultra-marathon courses of technical terrain or those requiring navigation. Nevertheless, the absolute CHO requirements for ultra-marathon racing are unclear. There is certainly a lower rate of CHO utilization during ultra-marathon relative to marathon. Laboratory data demonstrate that respiratory exchange ratio (RER) gradually decreases until the 8th hour of a 24 h treadmill run, and plateaus thereafter, reflecting a reduced rate of energy derived from CHO; moreover, this is congruent with a diminished running velocity [99]. As muscle glycogen diminishes, there is a compensatory increase in fat oxidation, with rates of 0.2–0.5 g·min^− 1^ typically observed during endurance exercise [100], and higher values of 1.0–1.5 g·min^− 1^ reported in a single subject after 6 h of running [101, 102]. The prolonged durations and slower relative running speeds that characterize ultra-marathon appear, therefore, to permit increased rates of fat oxidation for adenosine triphosphate (ATP) re-synthesis [100]. However, there is still a risk of glycogen depletion during ultra-marathon if work rate is too high, or if nutrition is poorly managed. Worthy of note is that extremes of both temperature and altitude will increase the absolute rate of CHO oxidation during exercise [102], and the nutrition strategy should accommodate these variations.

With respect to the absolute amounts of CHO and fats to be consumed during ultra-marathon, individual strategies vary greatly. There are reports that amateur runners contesting races of up to 70 miles (112 km) ingested CHO at a mean rate of 30 g·h^− 1^ [95]. In longer races (100 miles, 161 km), similar rates of CHO ingestion may be typical for slower finishers (31 ± 9 g·h^− 1^ [103];), both of which were lower than faster finishers (44 ± 33 g·h^− 1^); these data reinforce the notion of broad variance in the strategy used pending race pace or duration. Over the same distance, others report greater CHO intakes of 65.8 ± 27.0 g·h^− 1^ (range: 36–102 g·h^− 1^ [15];) compared to 41.5 ± 23.2 g·h^− 1^ for non-finishers (range: 13.8–83.8 g·h^− 1^). When expressed relative to body-mass, finishers consumed nearly double the amount of CHO than non-finishers (0.98 ± 0.43 versus 0.56 ± 0.32 g·kg^− 1^·h^− 1^). Similar values are reported in elite runners (71 ± 20 g·h^− 1^) during single-stage races [96]. Although current literature advocates CHO ingestion rates up to ~ 90 g·h^− 1^ for events > 120 min, particularly when using ‘multiple transportable carbohydrates’ containing glucose and fructose [104], such high rates of ingestion may be unrealistic for longer ultra-marathon races (> 6 h). Moreover, this rate of ingestion may lead to nutrient malabsorption and GI distress [105]. Worthy of consideration is that a CHO target of 90 g·h^− 1^ would necessitate a race diet almost exclusively comprising CHO (360 Kcal·h^− 1^) which is typically unsustainable given the greater preference for fat and salt that manifest in longer races.

With increasing race distance, a greater proportion of calories from exogenous fat may be critical for success [95]. Throughout a 100-mile race, finishers consumed a total of 98.1 ± 53.0 g of fat, which was approximately 5-fold greater than that of non-finishers (19.4 ± 21.1 g); moreover, when normalized for body mass and running velocity, this equated to a rate of fat ingestion that was three times greater in finishers (0.06 ± 0.03 versus 0.02 ± 0.02 g·kg^− 1^·h^− 1^ [15]). Collectively, these data suggest that successful completion of ultra-marathon likely requires a higher degree of tolerance to both CHO and fat intake (either as solids or fluids). Foods with a greater fat content are advantageous during racing in terms of caloric provision per unit of weight, and this is pertinent for minimizing pack weight when running self-sufficient. Moreover, foods with a greater fat content (see Table 4) often contain more sodium, which may help mitigate the risk of exercise-associated hyponatraemia.

**Table 4:** Example foods consumed by athletes^a^ during single-stage ultra-marathon (35–100 miles, 56–161 km)

Food suggestion/serve^b^	Energy (Kcal)	CHO (g)	PRO (g)	FAT (g)	Na^+^ (mg)	CHO	PRO	FAT	Na^+^
Sports drinks (50 g powdered serve)	186	46	0	0	255	✓			✓
Sports drinks (50 g) with added electrolytes (1 tablet)	186	46	0	0	505	✓			✓
Energy gels (40 g)	91	23	0	0	50	✓			
Energy gels with 30 mg caffeine (40 g)	90	23	0	0	40	✓			
Sports energy bar (55 g)	180	36	2	2	100	✓			
Homemade granola bars (30 g) – no added salt	140	18	3	7	0	✓			
Homemade oat bars with syrup (90 g) – no added salt	340	45	5	20	250	✓	✓	✓	✓
Dates (30 g)	89	20	1	< 1	0	✓			
Bananas (150 g)	135	30	2	< 1	10	✓			
Banana chips (30 g)	102	4	< 1	9	100			✓	
Boiled potatoes (100 g) – no added salt	173	26	3	6	10	✓			
Fruit/malt loaf (2 slices)	129	25	4	1	230	✓			
Watermelon slices (1 slice)	45	10	< 1	< 1	100	✓			
Spread-based (jam) sandwich – 1 sandwich	218	46	7	1	475	✓	✓		✓
Spread-based (peanut butter) sandwich – 1 sandwich	342	38	12	17	568	✓	✓	✓	✓
Oatcakes (3 portions)	135	17	3	5	300	✓		✓	✓
Meat pastry products (60 g)	189	15	12	5	400		✓	✓	✓
Beef jerky (25 g)	103	3	8	6	520		✓		✓
Chorizo (45 g)	207	1	11	18	1600		✓	✓	✓
Salami sticks (22.5 g)	113	< 1	5	10	900		✓	✓	✓
Sports protein bar (64 g)	238	23	20	11	300	✓	✓	✓	✓
Sports mass gainer bar (120 g)	453	58	30	13	50	✓	✓	✓	
MCT energy bar (45 g)	240	11	10	19	105		✓	✓	
Macadamia nut butter (1 sachet; 28 g)	215	4	2	22	28			✓	
Trail mix (50 g)	224	25	4	11	200	✓		✓	
Salted cashew nuts (50 g)	296	9	11	23	200		✓	✓	
Cheese bites (42 g / 2 portions)	140	0	10	12	320		✓	✓	✓
Salted potato chips (28 g / 16 chips)	150	15	1	9	150	✓		✓	
Green olives, medium (50 g / 15 olives)	75	3	0	6	285			✓	✓

^a^Examples taken from a survey of recreational to elite ultra-marathon runners (*n* = 12). ^b^Based on typical serving sizes. *MCT* medium chain triglycerides, *CHO* carbohydrate rich foods, *PRO* protein rich foods, *FAT* fat rich foods; Na^+^ = foods providing relatively greater amounts of sodium (> 250 mg). Amounts are typical serves, based on commercial brands for example purposes only, and will vary pending ingredients and additives. Athletes should consider individual tolerances and sensitivities. During single-stage races, recommended target ranges are: Energy = ~ 150–400 Kcal·h^− 1^; CHO = 30–50 g·h^− 1^; PRO = 5–10 g·h^− 1^; FAT = 1.1–17.7 g·h^− 1^; Fluid intake = 450–750 mL·h^− 1^; Sodium = > 575 mg·L^− 1^

#### Protein intake

Protein ingestion during racing is often neglected, for two possible reasons: i) protein plays a secondary role in energy metabolism under race conditions and athletes, therefore, prioritize the ingestion of CHO and fat; and ii) strategic ingestion of protein is difficult when runners rely solely on fixed checkpoints for the supply of energy/fluid and are, therefore, at the mercy of race organizers to supply foods with adequate protein. Nevertheless, it is plausible that protein ingested *during* an ultra-marathon would mitigate the ill-effects of muscle damage and/or positively influence energy metabolism. Indeed, finishers of a 100-mile (161 km) race had a significantly greater protein intake relative to non-finishers (131.2 ± 79.0 versus 43.0 ± 56.7 g) and, when expressed as a relative ratio per hour, race finishers consumed twice the quantity (0.08 versus 0.04 g·kg^− 1^·h^− 1^) [15]. Gastrointestinal distress and a lack of appetite in non-finishers may explain their lower overall intake.

Protein is likely an important component for prolonged endurance exercise because of the substantial proteolysis and muscle damage that can manifest before the conclusion of a race. In controlled studies, however, there are conflicting results. Protein co-ingested with CHO during 6 h of running and cycling improved net protein balance to a greater extent than the ingestion of CHO alone [106]. By contrast, when ultra-marathon runners were supplemented with 52.5 g of amino acids or a placebo prior to, and during, a 62-mile (100 km) race, there were no significant differences in markers of muscle damage or overall performance [107]. As such, the equivocal findings may result from the co-ingestion of protein and CHO, and/or differences in the exercise modality used between studies. Irrespective, nutrition strategies should be implemented that mitigate the consequences of prolonged protein abstinence, and a balance of macronutrients should be consumed.

A degree of self-sufficiency when racing may provide an opportunity for runners to follow a more bespoke nutrition strategy to better satisfy individual protein needs (see Table 4 for example foods). Protein-rich foods can be carried in running belts and/or backpacks and consumed ad libitum, but race organizers are also encouraged to provide high-protein options at checkpoints. Runners who are concerned that consuming calories from protein might compromise energy availability (i.e., by necessitating fewer calories from CHO and fat) might consider BCAA supplements (as liquid or tablets) as an alternative, particularly when the availability of protein-rich foods is limited. Where possible, ultra-marathon runners should strive to meet the typical dietary guidelines by consuming ~ 20–30 g of protein every 3 h [69].

#### The central fatigue hypothesis

Another means by which amino acid supplementation might provide an advantage during ultra-marathon racing is in offsetting central fatigue. Prolonged exercise increases the synthesis and metabolism of 5-hydroxytryptamine (5-HT; serotonin) in the brain, which is associated with lethargy, drowsiness, and reduced motivation [108]. Critically, tryptophan (the 5-HT precursor) competes with BCAAs to cross the blood-brain barrier [109], with the hypothesis that increasing the circulating concentrations of BCAAs might mitigate 5-HT accumulation, attenuate the seretonin:dopamine ratio [110], and potentially offset central fatigue. Indeed, athletes showed reduced effort perceptions when BCAAs were supplemented during submaximal cycle exercise performed in a glycogen-depleted state [111]. Moreover, when trained cyclists undertook several hours of exercise in the heat to exacerbate the central component of fatigue, BCAA supplementation prolonged time to exhaustion [112]. It is feasible that the role of BCAAs in offsetting central fatigue may be further pronounced during the extreme-distance ultra-marathons, the conditions of which are rarely replicated, and difficult to perform reliably, in a laboratory environment. The effect of BCAAs on central fatigue is far from certain, and further studies specific to ultra-marathon running are needed to elucidate the mechanisms that might underpin any beneficial effects.

#### Savory vs. sweet

A key consideration for the ultra-marathon runner should be the palatability of food (and fluid), particularly in longer races. Moreover, tastes and food preferences will likely change throughout the course of the race [113]. There are several reports of runners complaining of the unpalatability of sweet foods, particularly energy gels and sports drinks, both in the heat [114] and in ultra-marathons > 60 miles contested in thermoneutral environments [115, 116]. These data indicate that the aversion to simple CHO is not exclusively dependent on ambient conditions but is also influenced by race distance and/or duration. The mechanisms underpinning the proclivity for high-fat/salty foods are unclear, but it has been speculated that athlete food preferences are made to maintain a consistent chemical balance in the body [115]. In the aforementioned studies, runners tended to exhibit a penchant for savory food (i.e., flavoursome, non-sweet, and containing greater relative amounts of fat and salt) in the latter stages of ultra-marathon, thereby supporting the notion that changes in food preference may reflect nutrient inadequacies resulting from long-duration activity. An important consideration is to what extent one must rely on food provided by organizers at pre-determined checkpoints, given that the nature of such food is unpredictable and may be in limited supply. Accordingly, it is recommended that runners anticipate food availability, and carry their own food to more accurately fulfil their individual needs. Finally, race organizers are encouraged to provide a variety of foods at checkpoints (including a mixture of proteins, carbohydrates, and fats; see Table 4), and to publish in advance the list of foods to be served at feed-stations, so as to aid athletes in their race preparation. In longer races (> 50 miles / 80 km) that require athletes to skip multiple meals, organizers should consider providing at least one hot, calorie-dense meal served at a strategic point in the race. This will break the monotony associated with repetitive feed stations, and afford the runner an opportunity to mitigate caloric deficits that will likely accumulate.

#### Evidence statement (category C)

Athletes should follow a contemporary CHO-loading approach in the 48 h prior to racing in order to commence fully-replete. Calorie deficits during racing are expected but can be minimized by consuming 150–400 Kcal·h^− 1^, pending differences in body mass, race distance/pace, and individual gut tolerance.

#### Evidence statement (category C)

Calories should be consumed from a combination of protein (5–10 g·h^− 1^), CHO (30–50 g·h^− 1^), and fat; however, foods with greater fat content may be preferred in longer races.

#### Evidence statement (category D)

As race duration increases, runners tend to favor savory foods, likely reflecting energy and electrolyte insufficiencies.

### Offsetting dehydration

Thermoregulation during exercise is largely dependent on the mammalian sweat response to evoke evaporative heat loss. Insufficient fluid replacement, therefore, results in a net loss of body water, the main consequence of which is dehydration-induced cardiovascular drift; i.e., a reduction in plasma volume and a necessary increase in heart rate to maintain cardiac output [117]. The result is a diminished exercise capacity [118], and an increased risk of heat illness and rhabdomyolysis [118]. Dehydration may also diminish cognitive performance [11, 118] and increase perceived exertion [119]. All of the above may compromise performance and exacerbate the risk of injury and/or illness during ultra-marathon, particularly in arduous races, those requiring navigation, or those contested on technical terrain. Although dehydration can result from running in cold conditions due to a blunting of the thirst response, dehydration is more of a risk during races in hot and/or humid conditions when sweat rates are increased [120]. Moreover, consideration should be given to whether hot ambient conditions are dry or wet since the latter will compromise evaporative heat loss, increase fluid requirements, and increase the risk of heat illness.

Drinking-to-thirst is an acknowledged means of maintaining hydration during short-duration exercise (<90 min), when environmental conditions are cool, and/or when exercise intensity is low (e.g., < 60% V̇O_2_max) [121]. Moreover, this strategy is considered the most appropriate method of minimizing the risk of hypo- or hyper-hydration during ultra-marathon [16]. However, given that most athletes choose to consume electrolyte formulas by ingesting fluids, drinking-to-thirst may result in the under-consumption of sodium and other vital electrolytes. In long-distance ultra-marathons, the most common hydration plan is drinking according to an individualized schedule [122]. Moreover, finishers tend to consume fluid at a greater rate than non-finishers [92]. Mean fluid ingestion rates of ~ 0.5 L·h^− 1^ have been observed during a road ultra-marathon of 62 miles (100 km), with a broad range in the total volumes consumed (3.3–11.1 L) [123]. Slightly higher ingestion rates of ~ 0.75 L·h^− 1^ have been reported in races of 100 miles (161 km [92]). Collectively, the available data suggest that there are broad individual intakes among ultra-marathon runners, but that successful runners tend to meet the lower-limits of recommended values.

Fluid ingestion that results in diluted plasma sodium may be indicative that runners are not meeting their sodium needs [92]. Over-hydration, and the consequent dilution of plasma sodium, can have severe adverse effects on health (see *Exercise-associated hyponatraemia*), and there are case-reports of water intoxication in runners who aggressively rehydrate [124]. Runners contesting ultra-marathon should aim to consume 150–250 mL of fluid approximately every 20 min during exercise [31, 125], but fluid intake should be adjusted pending environmental conditions, race duration, work rate, body mass, the degree of fluid tolerance, and prior gut training. Individuals wishing to optimize performance should determine their individual sweat rates, in advance, under conditions which resemble competition (i.e., a similar exercise intensity, terrain, environment) [121]. An accessible means of estimating sweat rate is to measure nude body mass pre- and post-exercise; this will allow for an individualized fluid ingestion strategy.

#### Exercise-associated hyponatraemia (EAH)

Sodium is the major ion of the extracellular fluid and contributes to the generation of action potentials for muscle contraction, but it also has an important role in fluid retention [118]. Hyponatraemia, a potentially fatal condition of cell-swelling, is clinically-defined as a serum sodium concentration < 135 mmol·L^− 1^. Modest symptoms include headache, fatigue, and nausea, but can result in seizures and death in severe cases [9]. Two key, interrelated mechanisms are responsible for hyponatraemia: i) excessive sodium loss from the extracellular fluid resulting from a high sweat rate (e.g., while exercising in the heat) and prolonged sweating (e.g., during long-duration exercise); ii) aggressive hydration strategies using non- or low-electrolyte-containing fluids, which precipitate overload of the extracellular fluids, thereby diluting serum sodium [9]. Although the condition is rare, and individual susceptibility plays a role in prevalence, the earliest reported cases were observed in ultra-marathon runners and Ironman triathletes [9] (i.e., during ultra-endurance exercise), and the athletes most commonly developing symptomatic hyponatremia typically participate in distance running events of > 26.2 miles (> 42.2 km) [126].

In order to reduce the risk of hyponatremia during long-duration exercise, runners should consume sodium in concentrations of 500–700 mg·L^− 1^ of fluid [118]. Slightly greater amounts of sodium (and other electrolytes) will be required in hot (e.g., > 25 °C / 77 °F) and/or humid (e.g., > 60%) conditions when sweat rates are elevated; in such conditions, runners should target ~ 300–600 mg·h^− 1^ of sodium (1000–2000 mg of NaCl). If consumed in fluid, sodium concentrations greater than ~ 1000 mg·L^− 1^ (50 mmol·L^− 1^) should be avoided as this may reduce drink palatability [127]. Indeed, there is anecdotal evidence that effervescent (dissolvable) electrolyte tablets, and liquid electrolytes added to water, can compromise drink palatability, particularly during long races or those contested in the heat, thereby resulting in reduced fluid consumption. As such, capsules or tablets that can be swallowed whole are recommended, thus leaving water untreated. The amounts taken should also be offset against the sodium consumed from salt-containing foods, although it should be noted that it is unlikely that the recommended rate of sodium intake will be achieved from foods alone. In addition, the concentrations of some electrolytes (e.g., sodium) in many commercially-available electrolyte replacement products are insufficient to meet the recommended intakes. As such, runners are encouraged to pay close attention to the ingestion method and composition of their electrolyte formula.

Given the inherent risks associated with EAH, greater care should be taken to educate ultra-marathon runners on its deleterious consequences. For example, there are data to suggest that although sodium ingestion may help attenuate the likelihood of developing EAH, sodium intake is not sufficient for this purpose when simultaneous with excessive fluid ingestion [89]. As a result, runners sometimes adopt a low-volume drinking plan instead of increasing sodium intake congruent with their needs [122]. Such poor practice must be challenged, since it is possible to consume adequate amounts of both fluid *and* sodium during prolonged exercise, with sufficient practice.

#### Evidence statement (category C)

Fluid volumes of 450–750 mL·h^− 1^, or 150–250 mL every 20 min, are recommended during racing. Electrolyte concentrations (particularly sodium) from commercial products may not be sufficient for optimal hydration, especially in hot/humid conditions, and additional sources of sodium should be considered with the aim of ingesting 500–700 mg·L^− 1^.

### Gastrointestinal (GI) distress

A common cause of non-completion and/or reduced performance in ultra-marathon racing is GI discomfort or distress. A conservative estimate is that 30–50% of athletes experience GI-related issues during ultra-marathon [128], although values of 70–80% have been reported [129, 130]. The type, duration, and severity of symptoms vary on an individual basis, with upper GI-tract related issues (e.g., nausea, vomiting, heartburn) more common in longer races compared with complaints relating to the lower GI-tract (e.g., bloating, diarrhea) [115]. In a large cohort of males and females (*n* = 272) competing in the Western States Endurance Run (100 mile; 161 km), the majority of athletes (96%) experienced GI symptoms at some point during the race, particularly at the hottest and likely most challenging part of the course, with 44% indicating that GI issues negatively impacted race performance. Nausea was cited as the most common symptom likely to affect race strategy (reported in 60% of athletes) [130], perhaps due to the subsequent impact on the ability to ingest food and fluid.

The pathophysiology of GI distress during ultra-marathon training and racing is multifactorial, but is likely the result of reduced mesenteric blood flow [131, 132], leading to relative GI hypoperfusion [133]. This is often predicated by dehydration and/or increased core temperature, which can further compromise gastric emptying and paracellular transport [134]. An increased appearance of systemic lipopolysaccharides (LPS) from gram-negative intestinal bacteria may result from acute intestinal tight-junction protein disruption, thereby provoking an immune response, as well as endotoxin-mediated GI distress [134]. In one study, 81% of runners requiring medical attention at the end of a 56 mile (90 km) ultra-marathon (Comrades Marathon, South Africa) were reported to have LPS concentrations exceeding 100 pg·ml^− 1^ [135], with 81% reporting both upper- and lower-GI distress (nausea, vomiting, and diarrhoea). While such post-race endotoxin concentrations are considered severe in athletes, other researchers have noted a ‘bi-phasic’ endotoxin response in 68% of athletes competing in an Ironman triathlon, which corresponded with acute recovery phase cytokinemia [136]. This ‘low-grade endotoxemia’ may, in part, influence individual recovery responses during the short-term (<12 h) and chronic (>36 h) post-race period.

#### Strategies to minimize GI distress

Symptoms pertaining to exercise-associated GI distress are highly individualized and may be related to predisposition, intestinal microbiome activity (based on bacterial quantity and species diversity), and feeding tolerance [137]. The primary nutritional cause of GI upset during ultra-marathon is the high intake of CHO, particularly hyperosmolar solutions (e.g., > 500 mOsm·L^− 1^ and > 8% CHO concentration) [128]. Runners experiencing upper-GI discomfort were reported to have a greater energy and CHO intake than runners not experiencing symptoms [115]. This supports the notion that high rates of CHO ingestion, although being beneficial for race completion, might actually exacerbate symptoms of GI distress. In addition, strategies that could mitigate the likelihood of LPS release into the blood and, thus, endotoxin-associated symptoms, include limiting the consumption of saturated fat [138], avoiding the consumption of non-steroidal anti-inflammatory drugs (NSAIDs) [139], and maintaining an adequate water intake [139].

The use of ‘multiple transportable carbohydrate’ solutions (i.e., those containing glucose, fructose, and/or maltodextrin) has been shown in trained individuals to increase overall intestinal absorption, facilitate increased total CHO oxidation rates, and limit the degree of gut discomfort typically observed with single CHO solutions (e.g., fructose) [104, 140]. Although many ultra-marathon runners rarely rely solely on sports drinks for energy and/or CHO intake during racing, use of solutions with multiple transportable carbohydrates may be an effective short-term strategy to limit the likelihood of non-completion due to energy under-consumption. Recognizing the early onset of GI distress, and strategizing to maintain energy intake close to target values regardless, may be the key to managing some GI-related issues. Although counterintuitive, there may be some instances when eating regardless of nausea will give the most relief from such symptoms, especially when nausea is caused by hypoglycemia.

Prior race strategies that either ‘train the gut’ or include/omit some food groups may provide a solution to limit the negative impact of GI symptoms during racing. While ultra-marathon training may elicit progressive behavioral changes (e.g., greater confidence in trialing personalized nutrition strategies) and physiological adaptations (e.g., increased intestinal tight-junction integrity and enhanced immunological response to endotoxin release [135]), targeted nutrition strategies may confer a degree of individual benefit. It is apparent that well-trained athletes can tolerate higher intakes of CHO during running [128], and that habituation to a high CHO diet enhances total carbohydrate oxidation rates which may be important for sustained race performance [141] and reduced GI upset. Where symptoms of irritable bowel syndrome (IBS) are present, practicing a low FODMAP (fermentable oligosaccharide, disaccharide, monosaccharide and polyol) diet has been shown to reduce GI distress acutely [142, 143]. While responses to low FODMAP diets may be highly individual, strategic implementation (under guidance of a qualified nutrition professional) in the days preceding a race, or during training when acute symptoms occur, may confer GI support. Nevertheless, further research is warranted to confirm whether such benefits are applicable during sustained running.

Finally, the use of probiotic bacteria, particularly including the gram-positive genera *Lactobacillus* and *Bifidobacterium* species, has been shown to modify GI microbiota [144] and may provide an adjunct nutritional strategy in cases pertaining to acute GI disruption (e.g., GI dysbiosis, exercise-associated GI permeability). There is evidence of reduced GI symptom prevalence and severity following the administration of probiotics [145, 146] although benefits may be individualized and strain-specific. Recently, 4 weeks of supplementation with *Lactobacillus acidophilus* (CUL60 and CUL21), *Bifidobacterium bifidum* (CUL20), and *Bifidobacterium animalis subs p. Lactis* (CUL34) was shown to reduce GI symptoms, and may be associated with the maintenance of running speed in the latter stages of marathon [147]. Chronic multi-strain interventions have also been shown to reduce fecal zonulin levels by ~ 25% in endurance-trained athletes, attributed to improved GI epithelial integrity [148]. The inclusion of dietary prebiotic nutrients (e.g., fructooligosaccharides, inulin, pectin) may also play an important role in short-chain fatty acid production, which may support epithelial integrity (for review, see [149]). The use of pre/probiotics has, however, been contested [105] and, at present, there is limited evidence of a beneficial effect in ultra-marathon racing; as such, caution is recommended before implementing a new strategy.

#### Evidence statement (category B/C)

Symptoms of upper-GI distress, particularly nausea, are commonly reported during ultra-marathons, are a cause of non-completion, and are more prevalent in longer races.

#### Evidence statement (category C)

To mitigate GI distress, runners should avoid highly concentrated CHO, and minimize dehydration. When symptoms manifest, runners can slow their pace and decrease their calorie intake, although persistent intakes of < 200 Kcal·h^− 1^ should be avoided in longer races.

#### Evidence statement (category B)

Nutritional strategies should be practiced in training, well in advance of racing, to allow sufficient time for GI adaptations that optimize CHO absorption, and mitigate GI distress.

### Supplements and drugs

#### Caffeine

Caffeine is widely consumed as part of a normal diet, and there is clear evidence-for-efficacy regarding its ergogenic properties in a variety of sports [150–152], although the extent of the ergogenic effect is largely dependent on inter-individual genetic variance [153]. Caffeine works via two potential mechanisms: firstly, there is a centrally-mediated ergogenic effect, whereby caffeine blocks adenosine receptors in the brain and inhibits the binding of adenosine, resulting in improved cognitive function and concentration; secondly, caffeine potentiates intramuscular calcium release, thereby facilitating excitation-contraction coupling to increase muscle contractile function (for review, see [154]). Caffeine can cause a number of side effects, however, including GI distress, headaches, and anxiety [155]. Caffeine strategies should, therefore, be carefully planned and practiced in advance of competition. It should be noted that while there is some evidence that reducing habitual intake prior to competition might enhance caffeine sensitivity on race day [156], the hypothesis has been contested [157].

Caffeine has been shown to positively impact endurance performance [158], but there is a paucity of data on the use of caffeine during ultra-marathon. One of the only studies to assess the caffeine habits of ultra-marathon runners found that elite athletes contesting a 100-mile (161 km) single-stage race reported total intakes of ~ 912 ± 322 mg, spread over 15–19 h of running [96]. It is the stimulant properties that are likely to be most important for runners, particularly in races of > 24 h when sleep deprivation will affect performance and athlete safety. However, the dose response is not linear (i.e., larger caffeine doses do not necessarily confer greater performance), and moderate rates of ingestion are likely sufficient to optimize ergogenic gains [159]. A conservative strategy may also mitigate the likelihood of side-effects. While single boluses of ~ 4–6 mg·kg^− 1^ (280–420 mg for a 70 kg athlete) are common in short-duration activities, frequent dosing of this magnitude is not recommended. If frequent doses are to be taken during ultra-marathon, then lower (more sustainable) amounts (e.g., 1–2 mg·kg^− 1^; 70–140 mg for a 70 kg athlete) are more appropriate and safer over several hours. Importantly, caffeine has been shown to be effective when taken in the latter stages of endurance exercise [160]; accordingly, ultra-marathon runners are encouraged to target any caffeine intake for the latter stages of competition. Although there are no specific guidelines pertaining to caffeine intake during prolonged ultra-marathon, repeat doses of 50 mg·h^− 1^ are likely to be well-tolerated, principally reserved for night-running when circadian rhythms are likely to be affected. Individual sensitivity should, of course, be carefully considered, and strategies well-rehearsed. Finally, given the ergolytic and/or dangerous effects of caffeine overconsumption, athletes are advised to double-check their doses, ensure their intakes are congruent with the empirical data and safety guidelines, and give special consideration to the method of delivery (fluid vs. tablets vs. gum).

#### Medium-chain triglycerides (MCTs) and ketone esters

Although enhanced fat oxidation may be facilitated by nutritional ketosis (evoked via caloric restriction, carbohydrate restriction, or chronic high-fat diets), current evidence does not indicate an ergogenic effect when compared to diets that have a moderate-to-high CHO content. For example, exogenous fatty-acid supplementation (e.g., MCTs) has been proposed as a strategy to enhance aerobic metabolism through the rapid absorption and utilization of fatty acids (or converted ketone bodies). Animal models indicate a potential mechanistic benefit for the inclusion of MCTs to enhance mitochondrial biogenesis through both Akt and AMPK signalling, thereby enhancing endurance performance [161]. Nevertheless, controlled studies show limited impact of MCTs on fuel utilization during exercise when human subjects are in a low-glycogen or a glycogen-replenished state [162]. A further consideration is that, in order to mitigate the likelihood of GI distress during exercise, MCT oil should only be taken in relatively small amounts (i.e., < 30 g), and such low doses may have a negligible influence on fuel utilization [102] and endurance performance [163]. Nevertheless, there are anecdotal reports of MCT use by ultra-marathon runners, during both training and racing, which warrant further study.

More recently, novel ketone esters have been shown to optimize fuel utilization without the need of evoking ketosis via carbohydrate and/or caloric restriction. Within 60 min of ingestion, a 500 mg·kg^− 1^ ketone ester increased beta-hydroxybutyrate (D-βHB) concentrations to levels associated with nutritional ketosis (~ 3 mmol·L^− 1^), and increased intramuscular fat oxidation even in the presence of replete glycogen stores or when co-ingested with CHO [50, 164]. Moreover, such metabolic flexibility resulted in a significant (2%) increase in endurance performance [50], although this was during exercise lasting < 120 min. Performance benefits have, however, been repeatedly refuted [165, 166]; as such, despite the compelling mechanistic basis for ketone esters to facilitate ultra-marathon performance, there is currently no direct evidence to this effect, and further research is needed.

#### Vitamins and minerals

In general, studies have found no benefit of chronic vitamin and/or mineral supplementation on exercise performance [167, 168]. However, in a report on the supplement habits of 20 ultra-marathon runners, 30% of respondents reported taking multivitamins, and 20% reported taking vitamin C before races [169], although consumption rates as high as ~ 70% have been reported in small cohorts [170]. To date, only one study has assessed the effect of vitamin/mineral supplementation on ultra-marathon performance, finding that daily ingestion of multivitamins and minerals for ~ 4 weeks before competition did not result in statistically significant differences in performance time between supplement users and non-users (The Deutschlandlauf Marathon, Germany) [169]. Accordingly, there is insufficient evidence that multivitamin and/or mineral supplementation is beneficial for ultra-marathon, except in the instance of a clinically-determined, pre-existing nutrient deficiency or dietary insufficiency. Athletes should ensure that normal dietary intake is sufficient to provide an appropriate variety and quantity of micronutrients.

Given the substantial oxidative stress associated with ultra-marathon competition, isolated vitamin C has been hypothesized as a means of attenuating the high prevalence of post-race immunosuppression, although the data are conflicting. For example, a relatively high dose of vitamin C (1500 mg·d^− 1^) for 7 days prior to a 50 mile (80 km) single-stage race (The Umstead race; NC, USA) failed to induce any group differences in oxidative or immune responses, including lipid hyrdroperoxide and plasma interleukin (IL)-6 [171]. By contrast, a randomized, placebo-controlled trial by Peters et al. [172] reported a significantly lower prevalence of upper-respiratory-tract infection (URTI) in finishers of a 56-mile (90 km) single-stage race following daily ingestion of 600 mg of vitamin C, for 14 days post-race. Moreover, in a 31-mile (50 km) race, Mastaloudis, et al. [173] observed a significant protective effect against lipid peroxidation in runners who had been supplemented with antioxidants (α-tocopherol at 300 mg·d^− 1^, and ascorbic acid 1000 mg·d^− 1^) for 7 weeks prior. Accordingly, acute supplementation in the *immediate pre- or post-race period* may mitigate oxidative damage and immunosuppression that precedes URTI, although further research is needed to corroborate these findings and establish the effects of acute, in-task supplementation. Chronic, daily supplementation with antioxidants is not recommended due to the potential blunting effect on several aspects of exercise-induced physiological adaptation (for review, see [174]).

#### L-glutamine

L-glutamine is the most abundant amino acid in the body, with an essential role in lymphocyte proliferation and cytokine production [175]. In catabolic and hypercatabolic situations, L-glutamine can be essential to help maintain normal metabolic function and is, therefore, included in clinical nutritional supplementation protocols and recommended for immune-suppressed individuals [175]. Nevertheless, in terms of mitigating immunodepression after exercise, the available evidence is not sufficiently strong for L-glutamine supplements to be recommended for athletes (for review, see [176]). By contrast, there is emerging research that, in addition to probiotic use, L-glutamine may provide adjunct nutritional support for GI epithelial integrity [177]. In a recent study under controlled conditions, GI permeability (assessed via serum lactulose:rhamanose; L:R) was attenuated following demanding exercise performed at 30 °C when participants consumed a pre-exercise beverage containing 0.25 g·kg^− 1^ fat-free mass of L-glutamine compared with placebo. Furthermore, the authors highlighted a potential dose response, with higher concentrations (0.9 g·kg^− 1^ fat-free mass) further attenuating the L:R ratio. It has been proposed elsewhere that L-glutamine supplementation may be associated with heat-shock factor-1 (HSF-1) expression, providing a mechanistic link to GI integrity via regulation of occludin tight-junction proteins [178]. Further research is warranted with respect to L-glutamine supplementation in the context of ultra-marathon.

#### Analgesics and anti-inflammatories

To mitigate the extreme peripheral stress associated with competition, ultra-marathon runners commonly use analgesics including NSAIDs (Ibuprofen or aspirin), non-opioid analgesics (paracetamol), and compound analgesics (co-codamol) [179]. The prevalence of NSAID use among ultra-marathon runners is as high as 60%, with 70% of runners using NSAIDs during racing [180, 181]. There are several reports of attenuated exercise-induced muscle inflammation, circulating creatine kinase levels, and muscle soreness when NSAIDs were administered prophylactically before exercise [182, 183]. By contrast, a number of studies have found no effect of NSAIDs on analgesia or inflammation during exercise [184–188]. Notwithstanding, NSAID use can cause serious adverse effects on cardiovascular, musculoskeletal, gastrointestinal, and renal systems, all of which might be exacerbated by ultra-marathon running (for review, see [179]). There is an increased risk of GI-injury with NSAID use, and this may be exacerbated in long-distance runners (contesting marathon and ultra-marathon) who already exhibit a greater incidence of GI-bleeding [189–191]. Frequent prophylactic use of NSAIDs is also associated with increased risk of renal side-effects [192, 193], and concern has been expressed about a possible causative role of NSAIDs on exercise-induced hyponatremia [194]. Given the equivocal evidence-for-efficacy and the acute contraindications, NSAID use during ultra-marathon is strongly discouraged. Importantly, up to 93% of endurance runners are naïve to any contraindications of NSAID use [195], indicating the need for greater education in this respect. We thereby recommend race organizers to discourage NSAID use among their participants.

Non-NSAID analgesics (e.g., paracetamol) are not prohibited by The World Anti-Doping Agency (WADA), principally because they are not considered performance enhancing, *per se*, but rather performance *enabling*. This group of analgesics appears to be better tolerated than NSAIDs during competition; nevertheless, concealing symptoms of pain might facilitate and/or exacerbate injury, and the importance of afferent pain signals to indicate potential tissue damage cannot be underestimated. Caution is urged, therefore, against the frivolous and systematic use of analgesics for symptom-masking.

Finally, there is evidence that up to 15% of legal supplements are inadvertently or deliberately contaminated with illegal drugs, which remain in the system for several hours following consumption, and that would result in a positive test for banned substances [196, 197]. Accordingly, there is a growing need for greater batch-testing of supplements, and special consideration should be given when athletes are entering races that are overseen by anti-doping organizations. This will be critical in minimizing the risk of inadvertent positive tests.

#### Evidence statement (category A)

Caffeine is a potent stimulant that may be beneficial during racing, particularly in the latter stages of longer events (> 24 h), when sleep deprivation might attenuate performance and jeopardize athlete safety on technical terrain.

#### Evidence statement (category B/C/D)

Despite the potential efficacy of other ergogenic aids (e.g., ketone esters, MCTs, vitamins, etc.), there are limited data to support their use, and further research is warranted.

#### Evidence statement (category B/C)

Runners should abstain from NSAIDs (e.g., Ibuprofen, aspirin), due to multiple contraindications including increased renal loads that are already exacerbated during ultra-marathons. Analgesics may provide effective pain-relief, but conservative use is advised in order to avoid the inadvertent masking of serious symptoms.

## Summary

Ultra-marathon is a rapidly-growing sport contested by amateur and elite athletes the world-over. Due to its dynamic and complex nature, runners must endure myriad physiological stresses which can substantially impinge on both health and performance. This Position Stand highlights the nutritional considerations that are important for facilitating training adaptation, improving race performance, and mitigating the negative consequences of participation. These recommendations, as outlined in our evidence statements, should be considered by athletes and coaches, and may inform best-practice of those overseeing ultra-marathon events (i.e., race organizers and medics).

## Data Availability

Not applicable.

## Abbreviations

5-HT: 5-Hydroxytryptophan
AMPK: Adenosine-5′-phosphate- (AMP-) activated protein kinase
ATP: Adenosine triphosphate
BCAA: Branched chain amino acid
BF: Body fat
CHO: Carbohydrate
D-βhb: β-Hydroxybutyric acid
EAH: Exercise-associated hyponatremia
FODMAP: Fermentable oligosaccharide, disaccharide, monosaccharide and polyol
GI: Gastrointestinal
GLUT4: Glucose transporter 4
HSF-1: Heat shock factor 1
IL: Interleukin
ISSN: International Society of Sports Nutrition
LPS: Lipopolysaccharide
MCT: Medium chain triglyceride
NHLBI: National heart, lung, and blood institute
NSAID: Non-steroid anti-inflammatory drug
RCT: Randomized-controlled trial
RED-S: Relative energy deficiency in sport
RER: Respiratory exchange ratio
URTI: Upper-respiratory-tract infection
V̇O_2_max: Maximal oxygen uptake
WADA: World Anti-Doping Agency
